# The Degree of Segmental Aneuploidy Measured by Total Copy Number Abnormalities Predicts Survival and Recurrence in Superficial Gastroesophageal Adenocarcinoma

**DOI:** 10.1371/journal.pone.0079079

**Published:** 2014-01-16

**Authors:** Jon M. Davison, Melissa Yee, J. Michael Krill-Burger, Maureen A. Lyons-Weiler, Lori A. Kelly, Christin M. Sciulli, Katie S. Nason, James D. Luketich, George K. Michalopoulos, William A. LaFramboise

**Affiliations:** 1 Department of Pathology, University of Pittsburgh School of Medicine, Pittsburgh, Pennsylvania, United States of America; 2 Department of Internal Medicine, University of Pittsburgh School of Medicine, Pittsburgh, Pennsylvania, United States of America; 3 Department of Cardiothoracic Surgery, University of Pittsburgh School of Medicine, Pittsburgh, Pennsylvania, United States of America; CCR, National Cancer Institute, NIH, United States of America

## Abstract

**Background:**

Prognostic biomarkers are needed for superficial gastroesophageal adenocarcinoma (EAC) to predict clinical outcomes and select therapy. Although recurrent mutations have been characterized in EAC, little is known about their clinical and prognostic significance. Aneuploidy is predictive of clinical outcome in many malignancies but has not been evaluated in superficial EAC.

**Methods:**

We quantified copy number changes in 41 superficial EAC using Affymetrix SNP 6.0 arrays. We identified recurrent chromosomal gains and losses and calculated the total copy number abnormality (CNA) count for each tumor as a measure of aneuploidy. We correlated CNA count with overall survival and time to first recurrence in univariate and multivariate analyses.

**Results:**

Recurrent segmental gains and losses involved multiple genes, including: HER2, EGFR, MET, CDK6, KRAS (recurrent gains); and FHIT, WWOX, CDKN2A/B, SMAD4, RUNX1 (recurrent losses). There was a 40-fold variation in CNA count across all cases. Tumors with the lowest and highest quartile CNA count had significantly better overall survival (p = 0.032) and time to first recurrence (p = 0.010) compared to those with intermediate CNA counts. These associations persisted when controlling for other prognostic variables.

**Significance:**

SNP arrays facilitate the assessment of recurrent chromosomal gain and loss and allow high resolution, quantitative assessment of segmental aneuploidy (total CNA count). The non-monotonic association of segmental aneuploidy with survival has been described in other tumors. The degree of aneuploidy is a promising prognostic biomarker in a potentially curable form of EAC.

## Introduction

The last several decades have witnessed a substantial increase in the incidence of gastroesophageal adenocarcinoma (EAC) in the United States [1], [2]. The outcome and treatment strategy for EAC depends on the extent of local invasion and presence of regional or distant metastases at the time of diagnosis [3], [4]. Superficially invasive (EAC), because of the lower predicted risk of metastases relative to more locally advanced EAC, is potentially cured by esophagectomy or endoscopic resection [5], [6]. However, a subset of patients with superficial EAC develops regional and distant metastases and succumbs to their disease [7], [8]. Because of the broad range of potential treatment modalities [9] and clinical outcomes, superficial EAC requires accurate prognostication at the time of initial diagnosis when clinically aggressive tumors have the greatest chance of cure. Prognostic biomarkers are consequently more likely to impact the care of patients with superficial EAC.

Genomic instability contributes to malignant transformation by generating the clonal diversity that allows for the development of increased growth rates, invasion and metastasis in cancer cells [10]–[12]. Genomic instability and resultant aneuploidy is an early event in the pathogenesis of EAC. When detected in Barrett's esophagus it is a risk factor for progression to EAC [13]. Relative to other tumor types, EAC is known to exhibit a higher degree of chromosomal aneuploidy, characterized by loss and gain of whole chromosomes and shorter chromosome segments [14]. This deregulates genes in EAC by several mechanisms, including segmental amplification of oncogenes (e.g. EGFR, HER2, MET, KRAS, MYC); focal inactivating deletion of tumor suppressor genes (e.g. CDKN2A, FHIT); and point mutation of tumor suppressor genes (e.g. TP53) and accompanying copy neutral loss of heterozygosity [15]–[19]. Although previous reports have characterized genes involved in the pathogenesis of EAC by high throughput sequencing and array-based methodologies, none has focused on genomic instability in EAC and its potential prognostic significance. Moreover, none of these previous studies has specifically addressed the potentially curable subset of EAC that are limited to the superficial layers of the esophagus that may represent an early form of EAC.

Aneuploidy, when assessed by crude measurement of DNA content, is associated with worse prognosis in colon and lung cancer [20], [21], but there is conflicting data for EAC [22]–[24]. High density SNP array or array CGH can quantitatively assess segmental aneuploidy at high resolution. Recent studies have found that the total copy number abnormality (CNA) burden correlates with other measures of chromosomal instability in breast cancer cell lines [25] and is associated with poor prognosis in chronic lymphocytic leukemia and melanoma [26], [27]. However, the association between genomic instability and tumor behavior is complex. Experimental induction of severe chromosomal instability in cell lines can cause tumor cell death [28]. Furthermore, there is evidence in multiple solid tumors of a favorable prognosis associated with extreme levels of chromosomal instability [29]–[31].

To address the association of genomic instability with the clinical behavior of superficial EAC, we evaluated 41 tumors on the high density Affymetrix SNP 6.0 array to identify regions of CNA. This allowed us to quantify CNAs across the entire genome and assess the prognostic significance of segmental aneuploidy in each tumor. We also assessed the prevalence of gains and losses involving genes with a known pathogenic role in EAC to establish their prevalence in this potentially curable subset of EAC and identified several novel chromosomal regions harboring genes not hitherto implicated in the pathogenesis of EAC.

## Methods

### Clinical pathologic and survival variable definitions

This study was approved by the University of Pittsburgh Institutional Review Board with waiver of consent because the research involved the use of excess tissue obtained for routine treatment purposes and posed no more than minimal risk to subjects. The data were analyzed anonymously. The clinical and pathologic records were searched to identify superficial esophageal and gastroesophageal junction adenocarcinomas that were treated by esophagectomy between 1996 and 2010 without induction therapy. Barrett's esophagus was defined as esophageal intestinal metaplasia confirmed histologically in either pre-operative biopsy or in the esophagectomy specimen. Tumors location was classified as gastroesophageal junction (GEJ) or esophageal based on the 7^th^ edition AJCC criteria. Tumor specific pathologic variables were confirmed on pathologic review. Patient age and sex were obtained from the clinical record. Time to first recurrence was defined as the time from esophagectomy to first documented recurrence (locoregional or distant) and censored at the last clinical evaluation for recurrence. For overall survival, death was determined from review of the patient's clinical history as well as the social security death index and survival was censored at the time of last contact. Details of post-operative chemotherapy and/or radiation therapy were available in the medical record at our institution for 23 of 41 patients because many patients receive adjuvant therapy (when needed) at other institutions.

### Statistical analysis

Differences in categorical variables were assessed by chi-squared test or Fisher's exact test when appropriate. Differences in continuous variables were evaluated by Mann-Whitney U Test. Pearson's coefficient r or Spearman's rho was calculated to evaluate the correlation between continuous variables. Survival differences between groups were evaluated by comparing Kaplan-Meier survival functions with log rank test and Cox proportional hazards analysis. Significant differences were defined by p-value<0.05. All statistical tests were two sided. Calculations were performed using SPSS version 20 (IBM Corporation, Armonk, NY).

### Tissue selection

Superficial EAC were screened to identify tumors with adequately preserved, formalin fixed paraffin embedded (FFPE) tissue suitable for DNA extraction. Tumors exhibiting extensive autolysis were excluded. Tumors greater than 1 cm with high tumor epithelial cellularity were chosen for DNA extraction. Histologically benign lymph nodes between 0.5 and 1.0 cm were chosen as matched normal control tissue for each tumor due to the high DNA content of this tissue. They were histologically confirmed as benign on a minimum of three recut sections from the paraffin block. Ten 10 micron sections of tumor were macrodissected from glass slides guided by a serial H&E section to minimize admixture of normal tissue and ensure >75% tumor DNA content. Matched normal lymph node tissue was processed similarly from ten 10 micron sections.

### DNA isolation, hybridization and array processing

Genomic DNA from the FFPE tumor and matched FFPE normal samples was prepared for hybridization to the array using modifications of the procedures described by Thompson et al. and Teufferd et al. [32], [33] Genomic DNA was purified from formalin-fixed, paraffin embedded (FFPE) tumor and normal lymph node tissue according to a modified protocol for the QiaAmp DNA FFPE Tissue Kit (Qiagen, Valencia, CA). For each macrodissected specimen, tissue was placed into 2.0 ml Eppendorf tubes for deparaffinization through successive xylene and ethanol washes. Deparaffinized tissue was resuspended in Qiagen ATL buffer plus Proteinase K (300 ul) (>600 mAU/ml) followed by incubation (18 hours at 56°C) with shaking at 600 rpm in a thermomixer heat block (Eppendorph, Hauppauge, NY). Following tissue lysis and protein removal, samples were placed at 90°C for 1 hour to remove formalin crosslinking. Genomic DNA eluted from the QIAamp column was suspended in 53 µl of Buffer AE (Qiagen) and subjected to spectrophotometric analysis (NanoDrop, Wilmington, DE). Samples with absorption ratio of 260/280>1.8 were evaluated for DNA integrity using an Agilent Bioanalyzer 12000 DNA chip (50 ng/ul). Only samples with fragment sizes in the 1000 bp–3000 bp range (FU≥5) were included in subsequent microarray assays. Single nucleotide polymorphism analysis was performed using a modified version of the Affymetrix Genome-Wide SNP 6.0 protocol (Affymetrix, Sunnyvale, CA). Individual one microgram Sty I and Nsp I restriction digests (New England Biolabs, Ipswich, MA) were carried out and ligated with the matching adaptor (Sty or Nsp) provided in the Affymetrix 6.0 protocol. Twelve PCR reactions per sample were performed (6-Sty I digest/ligation reactions, 6-Nsp I digest/ligation reactions) followed by purification of PCR products using standard isopropanol precipitation (RT) followed by 70% ethanol precipitation on ice. DNAse 1 fragmentation was performed on 220 ug of pooled PCR product (110 ug STY and 110 ug NSP) and the fragmented DNA was biotinylated and end-labeled. Samples were then hybridized on Genome-Wide SNP 6.0 arrays for 18 hours at 50°C with rotation (60 rpm) in an Affymetrix Gene Chip Hybridization Oven (Model 640). The arrays were washed, stained and scanned according to the Affymetrix Genome-Wide SNP 6.0 protocol using the Affymetrix GeneChip Fluidics Station (Model 450), GeneChip Scanner 3000 7G, and GeneChip Command Console (v3.0) software.

Affymetrix Genotyping Console software (Affymetrix, Sunnyvale, CA ) was used to assess array data quality metrics (QC call rate and contrast QC). Affymetrix CEL and CHP files were transferred to Partek Genomics Suite (Partek Inc., St Louis, MO) for copy number analysis. Copy number was generated by comparing the hybridized intensities of each tumor array with the matched normal DNA sample from the same patient. Copy number measurements were smoothed based on local guanine-cytosine content using a 1-megabase window. The Partek segmentation algorithm was used to identify regions of significant variation from normal, consisting of a minimum of 20 genomic markers and p-value<0.0005, with signal to noise set at 0.7 and expected range 1.7 to 2.3 under the assumption that each tumor was diploid. Segmentation results are detailed for each case in Table S1. Human genome assembly hg19 was used to annotate each segment. All CEL and CHP files used for the study are accessible through the NCBI Gene Expression Omnibus (http://www.ncbi.nlm.nih.gov/geo/, accession number GSE49396).

### Analysis of segmental aneuploidy by total CNA count

After segmentation of individual cases, a database of all genomic segments harboring copy number abnormalities was used to calculate the number and size of all independent copy number abnormalities (CNAs) found in each individual tumor. An independent CNA was defined as a segment with copy number outside the pre-defined range of 1.7–2.3 that was not contiguous with an adjacent independent CNA of identical copy number. This measure is nearly identical to the total breakpoint count method previously described [25]. By this definition, adjacent segments with unequal copy number were defined as independent CNAs. We quantified the degree of segmental aneuploidy in each tumor by the total number of independent CNAs.

### Identifying candidate target genes in regions of recurrent CNA

We recognized candidate genes by identifying chromosome segments with extreme CNA (outside the range 1.474–3.025) in at least 3 of 41 cases. The boundaries of this range represent the 10th and 90th percentile copy number for all segments that fell outside the normal range. The lateral boundaries of target chromosomal regions were conservatively defined as regions with extreme CNA in at least 2 cases (provided the segment spanned a region of extreme CNA in at least 3 cases). Each target segment was mapped back onto the human genome (assembly hg19) manually using the University of California at Santa Cruz Genome Browser (http://genome.ucsc.edu) [34] in order to identify the candidate genes spanned by targeted segments. The frequency of gain or loss of candidate driver genes was determined by the frequency of extreme CNA involving any portion of the chromosome spanned by the consensus coding sequence of the candidate gene.

### FISH validation of copy number abnormalities for MYC and EGFR

Tumor samples (0.6 mm cores) from all 41 cases were arrayed in duplicate or triplicate on tissue microarrays as previously described [35], sectioned and co-hybridized with gene- and centromere-specific probes as previously described [36], We co-hybridized a 120 kbp SpectrumOrange-labeled DNA probe targeting the MYC region of chromosome 8q24 (Abbott Molecular, Des Plaines, IL) along with a SpectrumGreen-labeled chromosome 8 centromeric probe (CEP8, Abbott Molecular, Des Plaines, IL). We also co-hybridized a SpectrumOrange-labeled 303 kbp DNA probe targeting the EGFR region of chromosome 7p12 (Abbott Molecular, Des Plaines, IL) along with a chromosome 7 centromeric probe (CEP7, Abbott Molecular, Des Plaines, IL). The number of gene specific probe signals and centromere signals per tumor nucleus were counted in a minimum of 30 tumor cell nuclei to compute the average EGFR/CEP7, EGFR/nucleus, MYC/CEP8 and MYC/nucleus ratios. Amplification was pre-defined as EGFR/CEP7>2.0 or MYC/CEP8>2.0, following the guidelines for clinical detection of HER2 amplification in adenocarcinoma of the stomach and GE junction [37].

## Results

### Clinical and pathologic characteristics of the study population

Clinical and pathologic features of the study cohort are summarized in Table 1 and detailed for each case in Table 1. All 41 tumors were treated by esophagectomy without induction therapy in a single institution. The majority were male and over age 66. Forty percent of tumors were located in the esophagus with the remainder classified as GE junction adenocarcinomas. The large majority (85%) were encountered in a background of intestinal metaplasia (Barrett's esophagus) either in the pathologic specimen itself or in pre-operative biopsy. All but one tumor was staged T1, including 6 T1a (confined to the mucosa) and 34 T1b (invasion into the submucosa). One tumor was re-staged from T1 to T2 on pathologic review of recut levels of the tumor. Almost 40% of tumors were metastatic to one or more regional lymph nodes and one patient had a distant metastasis (stage M1) at the time of esophagectomy.

**Table 1:** Clinical and Pathologic Characteristics of the Study Population.

Clinical and Pathologic Characteristics		All Patients
		N (%)
**Sex**	**Female**	6 (14.6)
	**Male**	35 (85.3)
**Age, median (IQR)**		66 (60–74)
**Mass Location**	**Esophagus**	16 (39.0)
	**GE Junction**	25 (61.0)
**T Stage**	**T1a**	6 (14.6)
	**T1b**	34 (82.9)
	**T2**	1 (2.4)
**N Stage**	**N0**	26 (63.4)
	**N1**	10 (24.3)
	**N2 or N3**	5 (12.1)
**No. LN resected, median (IQR)**		23 (14–27)
**M Stage**	**M0**	40 (97.6)
	**M1**	1 (2.4)
**Tumor size (cm), median (IQR)**		2.5 (1.8–4.0)
**Tumor grade**	**WD or MD**	25 (61.0)
	**PD**	16 (39.0)
**Barrett's Esophagus**	**No**	6 (14.6)
	**Yes**	35 (85.4)

### Target identification heuristic identifies known and novel genomic targets of copy number gain and loss in EAC

All segments with at least one CNA falling outside the 1.7–2.3 range are listed in Table S2. We filtered this list to identify chromosomal segments targeted in at least three superficial EAC by extreme CNA falling outside the range of 1.474–3.025 (see Methods). Regions of CNA are more likely to be biologically significant when they show extreme copy number change and are identified in multiple tumors [38]. The genomic regions identified by our heuristic approach are listed in Table S3 and Table S4. Our results are validated by the fact that a large number of the targets of gain and loss have been previously reported in at least one of three large studies of chromosomal copy number changes in gastric and esophageal adenocarcinoma that examined over 100 tumors using hybridization array platforms [14], [16], [19].

We also report several novel targeted gains involving ERBB4, PDGFRA, CDH6 and PTPN11 (Table S3) and focal regions of copy loss overlapping MAML2, JAK2 and ERG (Table S4). Our heuristic approach identified recurrent, megabase size deletion regions spanning multiple genes on chromosomes 11q24-q25, 21q22.3 and 22q11.1-q11.21. These larger regions span multiple genes of potential biological relevance such as CHEK1 and ETS1 (11q24-q25), SUMO3 (21q22.3) and BID (22q11.1-q11.21) (Table S4). These putative targets require further validation and evaluation to establish their biological significance in EAC and exclude the possibility of false discovery inherent in the analysis of large datasets.

### Recurrent CNAs in superficial EAC frequently target genes known to be associated with gastroesophageal carcinogenesis

Although the heuristic criteria we employed detected a majority of previously characterized significant gains or losses in EAC, we specifically interrogated our data to determine the frequency of extreme CNA occurring in a list of candidate genes specified in two recent, large studies of gastric and esophageal adenocarcinoma using high density SNP arrays in order to detect rare events (involving <3 cases) in these significant regions (Table 2) [14], [19]. We found evidence of gains in 13 of 19 (68.4%) amplified genes and loss in 11/15 (73.3%) genes targeted by deletion that were reported by Deng et al. and Dulak et al. (Table 2).

**Table 2:** Prevalence of Copy Gains and Losses in Superficial EAC in Previously Reported Candidate Genes.

Cytoband	Previously Reported Candidate Gene(s)	Reference(s)	Present Study Frequency of CNA, N (%)
**Gains**			
**12p21.1**	**KRAS**	13,18	4 (9.8)
**18q11.2**	**GATA6**	13,18	4 (9.8)
**8p23.1**	**GATA4**	13,18	4 (9.8)
**19q12**	**CCNE1**	13,18	2 (4.9)
**7q21.2**	**CDK6**	13,18	4 (9.8)
**11q13.2**	**CCND1, FGF3, FGF4, FGF19**	13,18	8 (19.5)
**17q12**	**HER2**	13,18	8 (19.5)
**7p11.2**	**EGFR**	13,18	6 (14.6)
**8q24.21**	**MYC**	13,18	4 (9.8)
**6p21.1**	**VEGFA**	13	4 (9.8)
**7q31.2**	**MET**	13,18	4 (9.8)
**12q15**	**MDM2**	13	0 (0)
**7q34**	**EPHB6**	13	0 (0)
**6q23.3**	**MYB**	13	0 (0)
**1q21.3**	**MCL1**	13	0 (0)
**10q26.12**	**FGFR2**	13,18	0 (0)
**3q26.2**	**PRKCι**	13	0 (0)
**5p14.3**	**near CDH12**	18	1 (2.4)
**13q22.1**	**KLF5**	18	3 (7.3)
**Losses**			
**3p14.2**	**FHIT**	13,18	23 (56.1)
**16q23.1**	**WWOX**	13,18	10 (24.2)
**9p21.3**	**CDKN2A/B**	13,18	6 (14.6)
**5q11.2**	**PDE4D**	13,18	1 (2.4)
**20p12.1**	**MACROD2**	13	6 (14.6)
**4q22.1**	**FAM190A**	13	1 (2.4)*
**18q21.2**	**SMAD4**	13	6 (14.6)
**21q22.12**	**RUNX1**	13	6 (14.6)
**9p24.1**	**PTPRD**	13,18	4 (9.8)
**6q26**	**PARK2**	13	0 (0)
**4q35**	**CASP3**	13	0 (0)
**11q22.3**	**ATM**	13	0 (0)
**6p25.3**	**GMDS**	18	3 (7.3)
**13q14.2**	**RB1**	18	0 (0)
**8p23.1**	**CSMD1**	18	1 (2.4)

large deletion region.

In our cases, copy gain in HER2, EGFR and MET receptor tyrosine kinases (Table 2) were generally mutually exclusive. However, in one case we detected co-amplification of HER2 and EGFR and in one other case there was co-amplification of HER2 and MET. KRAS gains also occurred in one case with HER2 gain and in one case with MET gain. No case had evidence of copy gain in more than two of these genes. Interestingly, we also found evidence of focal KRAS loss in 3 (7.3%) cases. This was not observed in conjunction with gains of HER2, EGFR or MET. Overall, 18/41 (43.9%) cases had copy gain of at least one kinase (HER2, EGFR, MET and KRAS) capable of activating downstream MAP kinase signal transduction.

GATA4 and GATA6 are zinc finger transcription factors. GATA4 regulates gastric epithelial differentiation [39] and interacts with CDX2 to regulate intestinal gene expression [40] among other roles during embryogenesis. GATA6 plays a significant role in human pancreatic organogenesis [41], and is amplified and overexpressed in pancreatic cancer [42]. There is evidence that it functions as a lineage survival oncogene in esophageal adenocarcinoma [43]. RUNX1 is a transcription factor with frequent inactivating mutations in myeloid leukemia [44].

Cell cycle deregulation appears to be a dominant theme in the pattern of recurrent CNAs in superficial EAC, including recurrent amplification of CCND1, CCNE1 and CDK6 and frequent deletion of CDKN2A. Inactivation of genes known to play a role in sensing and responding to DNA damage is another recurrent theme in superficial EAC. Deletion of FHIT was the most common CNA detected (involving 23/41 cases). Loss of FHIT, a tumor suppressor that functions as a sensor of genotoxic stress, may confer resistance to and permit accumulation of DNA damage [45]. WWOX was the second most common deletion event (involving 10/41 cases). WWOX encodes a protein that interacts with p73 and p53 and regulates cellular response to genomic damage; expression is lost in many malignancies in addition to gastric and esophageal adenocarcinoma and it functions as a tumor suppressor [46]. FHIT, WWOX span fragile sites (FRA3B and FRA16D) that are frequently mutated in precursor lesions to EAC, including Barrett's esophagus and Barrett's associated dysplasia [15], [17], [47].

Although mutations in TP53 are among the most common mutations in esophageal adenocarcinoma based on whole exome sequencing [18], copy number abnormalities involving TP53 were not observed in our cases, a fact that has been previously noted in other reports [17].

### FISH validation of CNAs involving EGFR and MYC

**Figure 1:**
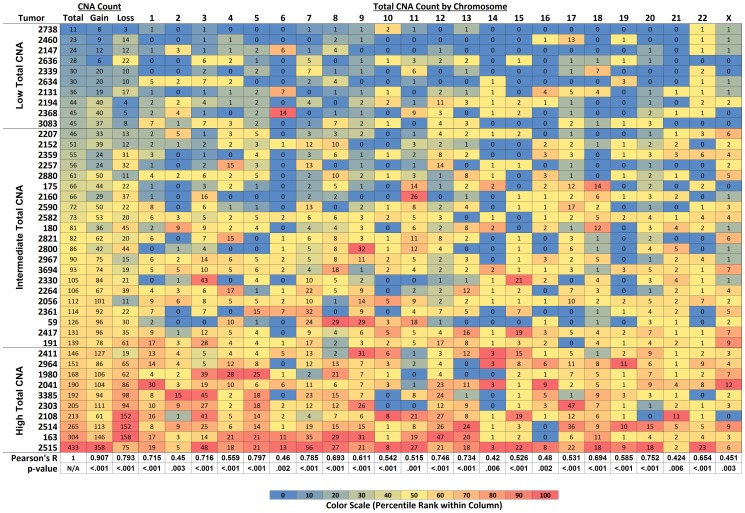
Representative EGFR and MYC FISH Results. FISH to determine EGFR and MYC copy number. Gene specific probes are labeled red (EGFR and MYC) while corresponding centromere probes (CEP7 and CEP8, respectively) are labeled green. (A) Tumor cells with a near normal 1∶1 ratio of EGFR/CEP7 and approximately 2 signals from each probe per cell. (B) High level EGFR amplification. EGFR amplification was detected as clusters of numerous red fluorescent signals which is a characteristic pattern caused by high EGFR copy number (see reference [57]) with variable chromosome 7 centromere copy number. (C) Tumor cells with a near normal MYC and centromere 8 copy number. (D) High level MYC amplification characterized by numerous MYC signals per cell and a high MYC/CEP8 ratio.

In order to confirm the SNP array assessment of copy number change in the tumors, we performed FISH using probes targeting EGFR and MYC with corresponding centromeric probes (CEP7 and CEP8, respectively). FISH for EGFR was successfully performed on 39 of 41 cases (Figure 1). There was a significant correlation between copy number by SNP array and EGFR/CEP7 as well as EGFR/nucleus ratios by FISH (Pearson's r = 0.926 and 0.861 respectively, p-value<0.001 for both). All three cases with high level amplification (EGFR/CEP7>15) by FISH showed copy gain by SNP array. There were two cases with low level amplification by FISH (EGFR/CEP7 = 2.0–4.0) that were not detected by SNP array (copy number within the normal range of 1.7–2.3). Of the six cases detected by SNP array (copy number>3.025), one case was unsuccessful by FISH, three were amplified at a high level, one case was borderline (EGFR/CEP7 = 1.89) with an average of over six copies of EGFR per nucleus. The last case was not amplified by FISH (EGFR/CEP7 = 1.13), possibly due to tumor heterogeneity given that the average copy number in the EGFR region by SNP array was 11.0.

All 41 cases were successfully evaluated for MYC amplification by FISH (Figure 1). There was a significant correlation between copy number by SNP array and both MYC/CEP8 and MYC/nucleus ratios by FISH (Pearson's r = 0.712 and 0.643, respectively; p-value<0.001 for each). Four of the five cases that were amplified by FISH also showed copy gain by SNP array (copy number>3.025). One FISH amplified case had lower level copy number gain (copy number = 2.7) in the MYC region by SNP array. Conversely, all four cases with copy gain by SNP array (>3.025) were amplified by FISH.

### Relative frequency and size of CNAs varies considerably in superficial EAC

EAC is known to exhibit greater aneuploidy than gastric (not including GE junction) and colonic adenocarcinoma as reflected in a greater number of arm-level and focal gains and losses [14]. We quantified the degree of aneuploidy in each tumor by evaluating the cumulative number of independent CNAs (copy number falling outside the normal range of 1.7–2.3). CNAs were not distributed evenly throughout the genome. They were most frequent in chromosomes 3 and 7 and least frequent in chromosomes 10, 14 and 19. Amplifications outnumbered deletions by a factor of 1.7.

**Figure 2:**
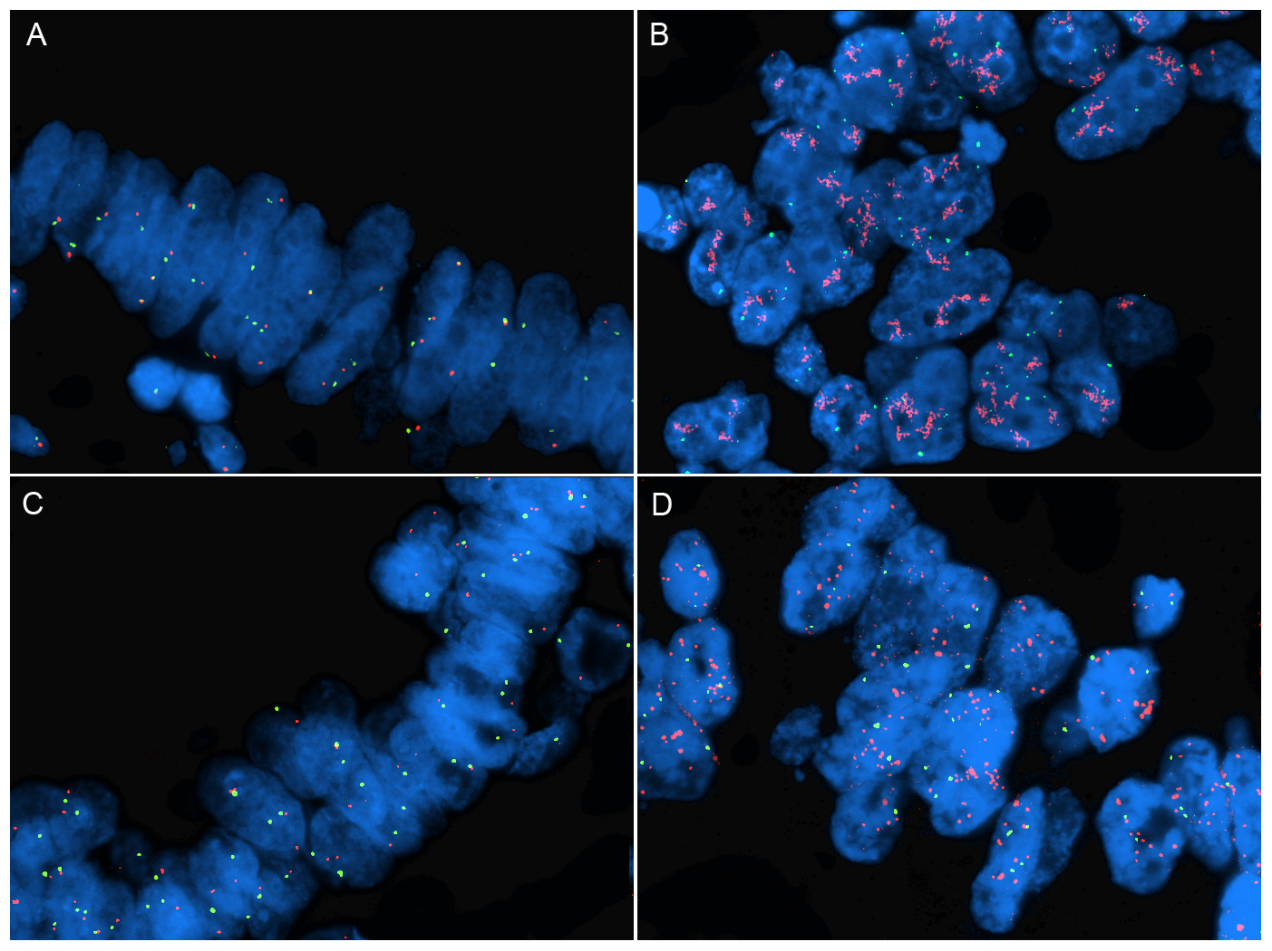
Heatmap Depicting the Correlation between Total CNA Count in Each Tumor and Total Copy Number Gains, Total Copy Number Losses and Total CNA Count by Chromosome. Cases are ordered from lowest to highest total CNA count down the left-most column. Lowest counts in each column are blue and the highest counts in each column are red as illustrated in the color scale below. In adjacent columns, the heatmap shows the correlation of total CNA count with total copy number gains, total copy number loss and total CNA count by chromosome. The heatmap illustrates that as total CNA count increases, the frequency of gains and losses increase and the frequency of CNA counts tends to increase throughout all chromosomes. Correlation coefficients for each column with total CNA count (Pearson's r) are listed below with the corresponding p-values.

**Figure 3:**
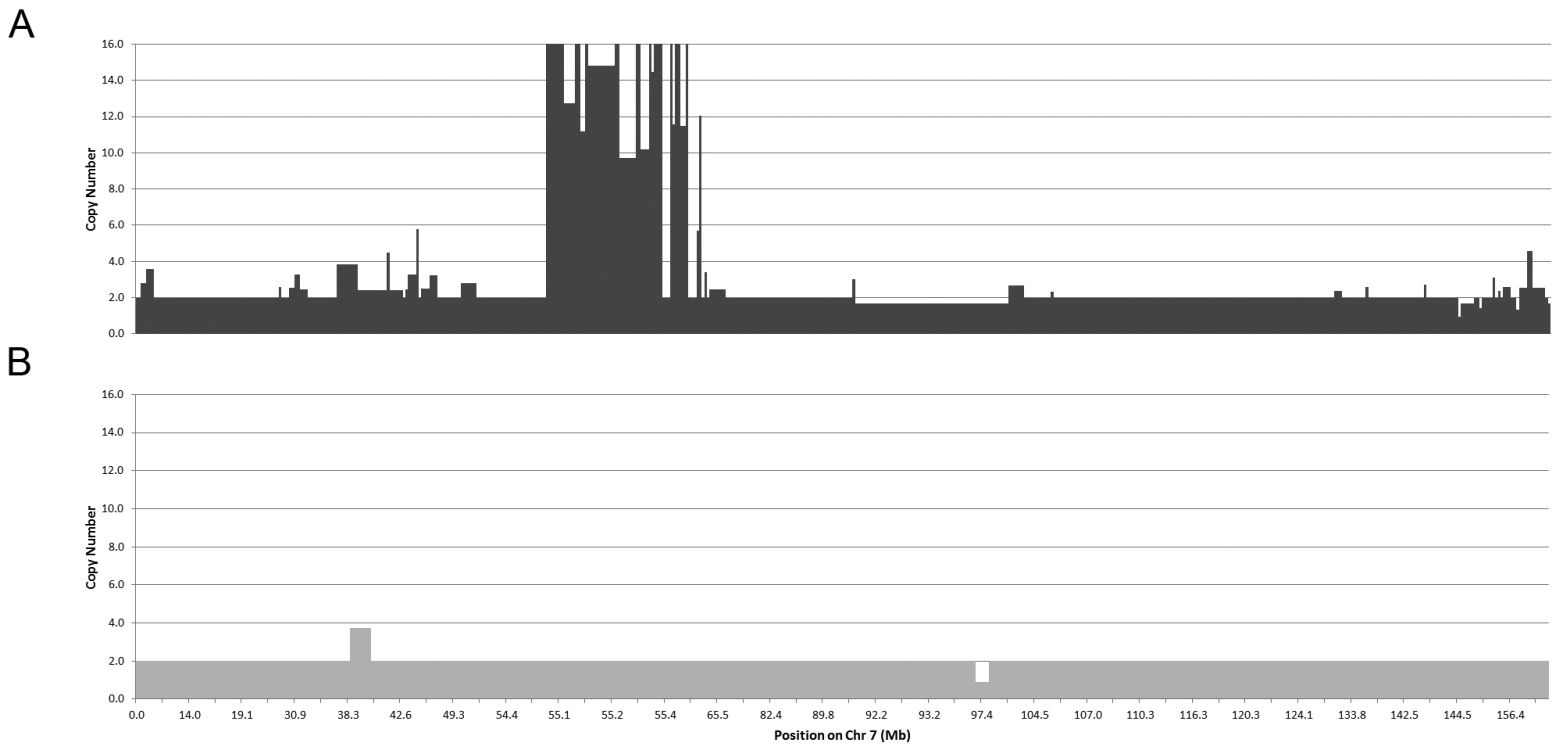
Schematic Illustration of Copy Number Abnormalities in Two Tumors Representing High and Low CNA Count and a Spectrum of Genomic Complexity. For both A and B, the horizontal axis represents position of segments along chromosome 7 (in Mb) and the vertical axis represents estimated copy number (truncated at 16). (A) Tumor 2515 (dark grey) has a large number of independent CNAs on chromosome 7 (N = 56), including a complex copy number gains at ∼55 Mb (region of EGFR) as well as other gains and losses throughout the chromosome. (B) By contrast, tumor 2634 (light grey) has few independent copy number changes (N = 2, gain at ∼39 Mb and loss at ∼97.4 Mb). For this illustration, copy numbers in the normal range of 1.7–2.3 were assigned a value of 2.

The frequency of CNAs ranged from 11 to 433 per tumor (Figure 2) with a median of 82 (IQR, 46–139), indicating a wide variation in genomic complexity in superficial gastroesophageal adenocarcinoma. Individual chromosomes also showed considerable variation among different tumors (Figure 2 and Figure 3). As expected, there was a strong correlation between total CNA count and total CNA count for gains or losses alone (Figure 2). The heat map (Figure 2) illustrates that tumors with high total CNA counts tend to have elevated CNA counts throughout the genome, evidenced by CNA counts above the median in the majority of their chromosomes. However, the correlation between total CNA count (including all chromosomes) and individual chromosomal CNA counts was better for chromosomes with relatively high CNA counts than those with low CNA counts (Figure 2).

Among the 4394 CNAs identified in the entire data set, the size of CNAs varied from sub-kilobase focal events to 155 Mb arm-level events. When grouped by size, 1.6% were <1 kb; 20.2% were 1–10 kb; 35.1% were 10–100 kb; 17.9% were 100 kb-1 Mb; 17.9% were 1–10 Mb; and 7.2% were >10 Mb. The median size of CNAs as we have defined them was 41.3 kb (IQR, 11.9 kb–1.04 Mb). The size of CNAs varied from tumor to tumor with the median size in a given tumor ranging from 7.4 kb to 266 kb. Four cases lacked any CNAs greater than 10 Mb; two of these had CNAs distributed throughout all smaller size ranges up to 10 Mb and the other two cases had >90% of CNAs less than 100 kb.

### Total CNA count is not associated with chromosome 7 or 8 copy number by FISH; FHIT deletion or WWOX deletion

We also evaluated whether total CNA count was associated with chromosome 8 or chromosome 7 copy number (based on centromeric FISH) as an independent, but indirect assessment of tumor ploidy. Polyploidy could reduce the detection of single copy gains and losses by SNP array [48] and possibly explain low CNA counts. We separated tumors into three groups: high CNA count (highest quartile), low CNA count (lowest quartile), intermediate CNA count (cases in the middle 2 quartiles). There was no difference among CNA count groups in the average chromosome 8 copy number by FISH (2.0, 2.2 and 2.1 in the low, intermediate and high CNA groups, respectively; p = 0.368, Kruskal-Wallis ANOVA), nor in the in the average chromosome 7 copy number by FISH (2.3, 2.7 and 2.9 in the low, intermediate and high CNA groups, respectively; p = 0.345, Kruskal-Wallis ANOVA). Lastly, there was no significant correlation between chromosome 7 or 8 CNA count and chromosome 7 or 8 ploidy by FISH (data not shown). These data suggest that tumor ploidy does not significantly alter the total CNA count.

FHIT and WWOX genes play a role in regulating response to DNA damage and mutations in these two genes might be more common in tumors with elevated CNA counts. We found that 40% of tumors in the low CNA count group had FHIT deletion, compared to 62% and 60% of intermediate and high CNA count cases, respectively (p = 0.600, Fisher's exact test). Likewise, we found that 10% of low CNA count tumors had WWOX deletion compared to 29% and 30% of intermediate and high CNA count tumors, respectively (p = 0.629, Fisher's exact test). Similarly, the mean CNA count among cases with FHIT mutation or WWOX mutation was not significantly different from cases lacking these mutations (data not shown, Mann-Whitney U Test p>0.05 for both).

### Total CNA count does not correlate with data quality metrics

Formalin fixation, which increases hybridization noise and reduces SNP array data quality relative to high quality frozen tissues [32], [33], could influence relative CNA counts. There was no correlation between total CNA count and standard array data quality metrics (QC call rate and contrast QC), indicating that the differences in CNA count were not an artifact caused by technical noise (Table S5).

### Total CNA count is associated with prognosis in superficial EAC

In the population of 41 cases, there were 19 deaths and 11 documented recurrences with a median follow up interval of 46 months (range 2.2–104 months). Two patients died within 3 months of surgery of post-surgical complications and were excluded from the analysis of overall survival. Recurrences were diagnosed at 16.5 months post-surgery on average (range 7–27 months). All 11 patients with tumor recurrence died.

**Figure 4:**
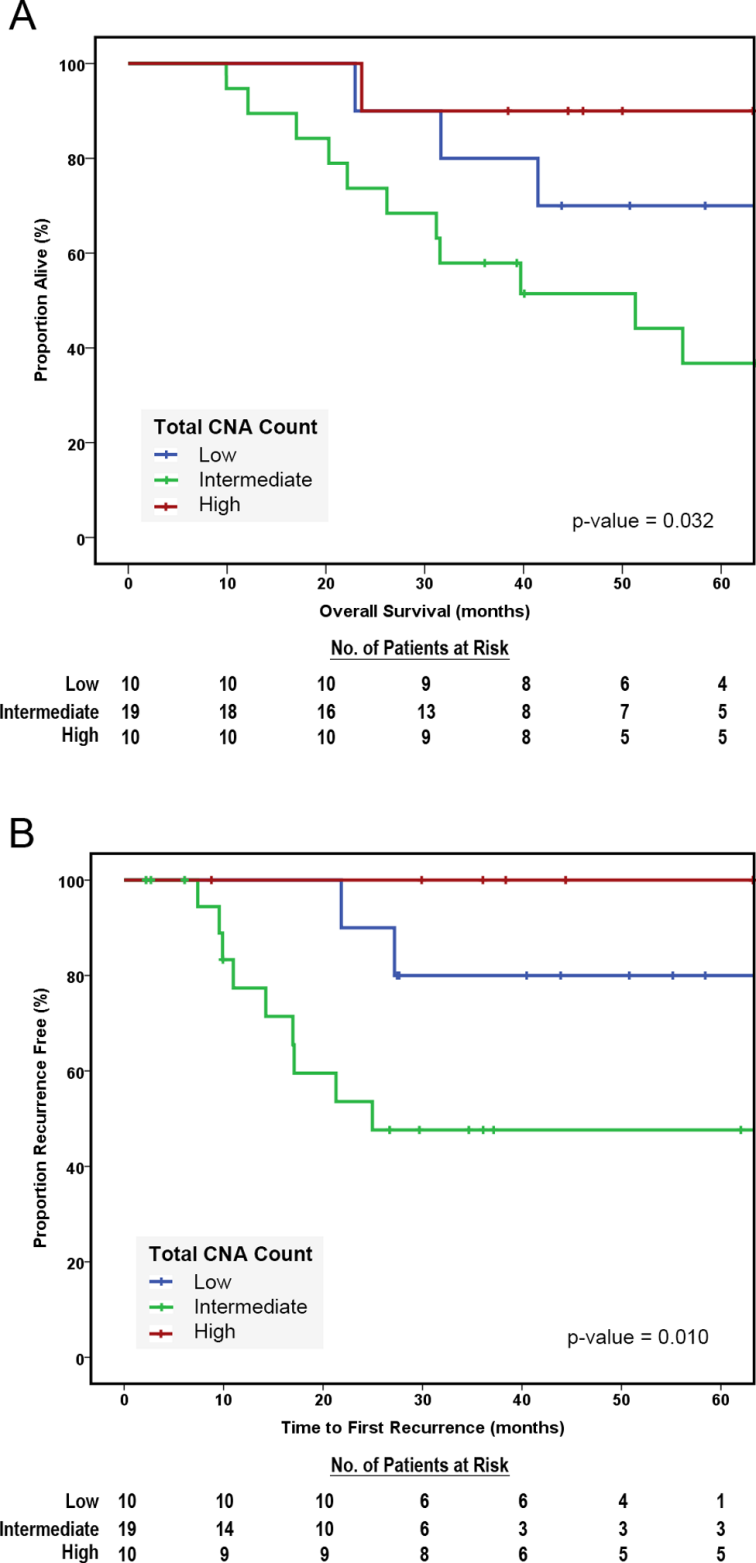
Overall Survival and Time to First Recurrence for Superficial EAC Stratified by Total CNA Count. (A) Patients with intermediate total CNA counts had significantly worse overall survival than patients with low or high total CNA counts (log rank p-value = 0.032). (B) Similarly, patients with intermediate total CNA counts had significantly shorter time to first recurrence than those with low or high total CNA counts (log rank p-value = 0.010).

We evaluated the prognostic significance of total CNA count in the three groups (low, intermediate and high) based on CNA count. The Kaplan-Meier survival curves reveal that patients with tumors characterized by intermediate CNA count had significantly worse overall survival outcomes than those falling into the highest or lowest quartiles (p-value = 0.032, Figure 4A). This same pattern was observed in time to first recurrence which is a better indicator of aggressive tumor behavior in superficial cancers (p-value = 0.010, Figure 4B).

The CNA count groups had no significant differences with respect to known pathologic prognostic variables (TNM stage, angiolymphatic invasion, tumor size, tumor grade, number of lymph nodes resected, Table 3). Multivariate analysis was performed to adjust for confounding prognostic variables. On univariate analysis, intermediate total CNV count was an adverse prognostic factor (HR 3.7, 95% CI 1.3–10.7) and remained a 3.4-fold increased hazard of death (95% CI 1.1–10.4) after adjusting for other borderline or significant (p-value<0.2) predictors of survival (N stage, angiolymphatic invasion and tumor size). Similarly, it was associated with a 7.3-fold increased risk of recurrence and remained a significant predictor of time to first recurrence (HR 7.3, 95% CI 1.5–34) after adjusting for angiolymphatic invasion (the only other variable with a borderline association with recurrence, p-value<0.2). Factors evaluated in the multivariate analyses included patient age, N stage, angio-invasion, tumor size, tumor grade and number of lymph nodes resected. We had data on adjuvant therapy for only 23 of 41 cases. In these 23 patients, administration of post-operative chemotherapy and/or radiation therapy was not a significant prognostic factor (HR 1.423, 95% CI 0.3–5.9) with respect to overall survival. The lack of complete data precluded inclusion of this variable in the multivariate analysis.

**Table 3:** Total CNA Count in Relation to Potential Pathologic Prognostic Variables.

		Total CNA Count		
		Low	Intermediate	High
**T stage** *	**T1a**	2 (20%)	3 (14.3%)	1 (10%)
	**T1b-T2**	8 (80%)	18 (85.7%)	9 (90%)
**N stage** *	**N0**	8 (80%)	12 (57.1%)	6 (60%)
	**N1+**	2 (20%)	9 (42.9%)	4 (40%)
**M stage** *	**M0**	10 (100%)	20 (95.2%)	10 (100%)
	**M1**	0 (0%)	1 (4.8%)	0 (0%)
**Grade** *	**WD-MD**	6 (60%)	12 (57.1%)	7 (70%)
	**PD**	4 (40%)	9 (42.9%)	3 (30%)
**Angioinvasion** *	**no**	8 (80%)	12 (57.1%)	7 (70%)
	**yes**	2 (20%)	9 (42.9%)	3 (30%)
**T size (cm), mean** †		2.26	3.30	3.03
**No. LN resected, mean** †		23	24	21

For categorical variables, none of the differences between groups was statistically significant when comparing across the three groups nor in pairwise comparisons with low CNA count (p-value>0.05, Fisher's exact test).

The difference in tumor size and mean number of resected lymph nodes was not significant across the three CNA count groups (p-value>0.05, Kruskal-Wallis one way ANOVA), nor in pairwise comparisons (p-value>0.05, Mann-Whitney U Test).

## Discussion

Although the spectrum of sequence-level and chromosome-level mutations that contribute to the pathogenesis of EAC are increasingly well characterized [14]–[18], no previous study has explored the clinical significance of chromosomal instability in detail. In this study, we have chosen to focus on superficial EAC because of the essential importance of prognostic information for optimizing therapy. In superficial EAC, we find that extreme low or high CNA burden connotes a relatively favorable prognosis in comparison to intermediate levels. Although we focus on superficial EAC, our results are likely to be generalizable because we saw significant similarities between superficial EAC and published results from unselected EAC with respect to patterns of recurrent gains and losses.

In our analysis of CNAs by high density SNP array, we see a 40-fold variation in the total number of CNAs detected in the tumor genome of superficial EAC. This suggests that there are significant differences between individual tumors in pathways that regulate chromosomal replication, segregation and repair of DNA damage. Chromosomal instability is a reflection of the inability of tumor cells to maintain a stable genome and is measured by directly evaluating intratumoral heterogeneity or chromosomal changes in tumor cell populations over time [49]. SNP arrays capture a static, aggregate image of a population of tumor cells, but give a more detailed portrait of arm level CNAs and segmental CNAs present in the genome. Nevertheless, the total CNA count by SNP array has been shown to correlate with direct measures of chromosomal instability and tumor ploidy in colorectal cancer cell lines [25]. In our analysis of segmental variation in copy number, we did not evaluate tumor ploidy, *per se*. Tumor ploidy might alter the estimated magnitude of copy number changes relative to matched normal cells, but this would not affect the total number of segmental CNAs. However, polyploidy would be expected to reduce the detection of single copy changes [48]. We evaluated chromosome 8 and chromosome 7 copy number by FISH using centromeric probes and found no evidence of higher centromere counts per nucleus among tumors with low CNA counts. If anything, increased ploidy appeared to be more common among tumors with high CNA counts—suggesting that we may be underestimating the magnitude of difference between tumors with low CNA counts and those with high counts. The actual CNA counts and CNA size estimates observed will also vary based on segmentation parameters, but relative differences in CNA should persist.

Chromosomal instability and aneuploidy are most often associated with poor prognosis in cancer, a generalization that is true for multiple cancer types [49]. However, there is evidence suggesting a more complex relationship between genomic instability and clinical behavior. One recent study using the CIN70 index [50] to measure chromosomal instability, found a paradoxical relationship between CIN70 scores and survival in estrogen receptor negative breast cancer, ovarian cancer, squamous carcinoma of the lung and gastric adenocarcinoma [30]. Tumors with the highest quartile CIN70 score had significantly better prognosis than tumors in all three other quartiles. Tumors in the third quartile had the worst prognosis. Our findings are similar, in that superficial EAC with high total CNA counts had favorable prognosis compared to those in the middle quartiles which had significantly worse survival outcome. Another study of serous ovarian carcinomas computed a total aberration index (TAI) from copy number profiles obtained from array CGH or SNP array platforms [31]. The TAI represents a weighted average copy number across the entire genome. Patients with TAI above the median had significantly better overall and progression free survival than those with TAI below the median. They did not report quartiles. The prognostic significance of TAI was also shown to be independent of age, tumor grade and tumor stage in their multivariate analysis.

Variation in the degree of aneuploidy not only has prognostic significance, but it has potential therapeutic implications. On one hand, chromosomal instability has been shown to facilitate the acquisition of drug resistance in tumor cells [25]. On the other hand, experimentally induced chromosomal instability has been shown to sensitize tumor cells to taxol, an anti-mitotic drug that inhibits microtubule formation [51]. Tumors with BRCA1 deficiency that compromises homologous recombination of DNA double-strand breaks have high levels of genomic instability and are sensitive to poly-ADP ribose polymerase (PARP) inhibitors and DNA cross-linking compounds [52]. Evaluation of genomic instability may identify EAC that are sensitive to agents that exploit deficiencies in DNA repair or maintenance of structural and numerical chromosomal integrity. Given the small number of patients with available data on post-operative chemoradiotherapy (23 of 41), we were unable to explore this question in our study.

Our analysis of CNAs in superficial EAC confirmed many gains and losses targeting receptor tyrosine kinases, cell cycle regulatory proteins, and lineage specifying transcription factors. Although superficial EAC is primarily treated by surgical resection, selective inhibitors that target HER2, EGFR, MET and KRAS—one or more of which we found to be amplified in approximately 40% of superficial EAC—are plausible therapeutic targets for recurrent superficial EAC. Amplification of FGFR2 has been reported to occur in 5–10% of gastric and esophageal adenocarcinomas by SNP array analysis [14], [19], but we did not see evidence of copy number gain in our cohort. Nor did we see copy number gains involving MDM2, EPHB6, MYB, MCL1 or PRKCι, nor losses in PARK2, CASP3, ATM and RB1. We did see recurrent CNAs at genomic fragile sites (FHIT, WWOX, MACROD2 and DMD) as previously described [47]. WWOX and FHIT are tumor suppressor genes that are mutated in a wide range of cancers and could result in greater genomic instability given their roles in detecting genotoxic stress and regulation of the cellular response to genomic damage [45], [46]. Although deletion events affecting WWOX and FHIT were marginally more common in tumors with intermediate or high CNA counts (compared to low), the differences were not statistically significant across all three groups. Hence, we cannot suggest a causal role for these fragile site deletions in the overall level of segmental aneuploidy. The study was not sufficiently powered to evaluate the prognostic significance of copy gain or loss of individual genes due to the low prevalence of most of the CNAs targeting biologically significant genes.

On a qualitative level, amplification events involving HER2 and EGFR were characteristically high copy number with variation in copy number throughout the amplified segment (as depicted in Figure 3). We cannot exclude differences in attenuation curves of neighboring SNP probes as an explanation for this phenomenon [53], but other mechanisms could account for this, such as successive somatic DNA alterations or chromothripsis [54], Chromothripsis is estimated to occur in approximately 2% of cancers, including esophageal carcinoma [55]. It is characterized by multiple genomic alterations involving random, but clustered copy number changes of similar size and alternating copy number profile, often resulting in loss deletion of genomic material. The copy gains in EGFR and HER2 did not fit this typical profile. A survey of our data did not locate regions of likely chromothripsis. However, other methods (e.g. paired end sequencing) are better suited to identify the characteristic sequence inversions and rearrangements of segment order that occur as a result of chromothripsis [54].

Other regions of recurrent CNA merit further investigation due to the potential role in the pathogenesis of EAC. For example, the region of chromosome 12q24.13 spanning the PTPN11 gene was amplified in approximately 17% of cases (Table S1). PTPN11 encodes the protein tyrosine phosphatase Shp2. It is an intriguing candidate because PTPN11 is mutated in Noonan and LEOPARD syndromes and activating mutations have been implicated in the pathogenesis of leukemia while inactivating mutation promotes the development of hepatocellular carcinoma [56].

Our study has limitations, including the retrospective design, the relatively small size of the study cohort compared to the largest studies and our use of FFPE tissue. As a retrospective study, patients did not receive rigorously standardized treatment and follow-up. We attempted to control for potential confounding variables in our analysis of clinical outcome, but we were unable to control for the effects of post-operative chemoradiation treatment. Although the number of cases in our study did not allow us to evaluate the prognostic significance of individual CNAs, ours is a large cohort of superficially invasive EAC and we believe that our data make a positive contribution to the characterization of the prevalence of CNAs and the potential clinical significance of segmental aneuploidy in a generally understudied disease. Lastly, to study the superficially invasive subset of EAC, we required FFPE specimens in the clinical archive. There is an inherent reduction in data quality associated with the use of FFPE specimens in comparison to high quality frozen samples. Nevertheless, FFPE tissue represents a much larger repository of clinically annotated samples. Based on prior reports, reliable copy number data can be obtained from FFPE samples using a variety of SNP array platforms, in spite of reduced data quality [32]. Supporting the validity of our data is the fact that we identified a large majority of recurrent copy gains and losses that have been previously identified in esophageal and gastric cancer. The prevalence of these CNAs in our samples was similar to what has been reported. The reduction in data quality due to formalin fixation would be expected to influence detection of CNAs, but we saw no correlation between standard data quality metrics and the number of CNAs to suggest that this was a major factor in total CNA count variation. Still, validation of the results in cohorts of more advanced EAC as well and evaluation of segmental aneuploidy and chromosomal instability as a marker of response to chemotherapy or radiation therapy will be of interest.

Our study highlights the potential clinical utility of genome wide analysis of copy number changes. First, it allows for the simultaneous evaluation of multiple potential therapeutic targets that are defined by copy gain or loss. This will become increasingly relevant as clinical trials evaluate the efficacy of targeted therapeutic agents in addition to trastuzumab for the treatment of HER2 amplified gastroesophageal adenocarcinomas [37]. Of course, SNP array technology may not ultimately prove to be the ideal platform because of the inability to interrogate mutations that occur at a smaller scale (i.e. at the sequence level). Second, the quantitative assessment of CNAs can be used to determine the pattern (whole chromosome versus segmental) and the extent of aneuploidy. Aneuploidy and genomic instability are promising prognostic markers. Future studies should address whether the degree of aneuploidy or genomic instability can predict response to therapies that target cells based on their capacity to respond to DNA damage. Prognostic and predictive biomarkers are needed for superficial EAC in order to optimize therapeutic outcomes for this potentially curable form of cancer.

## Supporting Information

Table S1 **Clinical and Pathologic Data for All Tumors.**
(XLSX)

Table S2 **Estimated Segmental Copy Number for Each Tumor in Chromosomal Segments with Abnormal Copy Number in at Least One Tumor.**
(XLSX)

Table S3 **Regions with Copy Number Gain (CN>3.025) in Three or More Tumors.**
(DOCX)

Table S4 **Regions with Copy Number Loss (CN<1.474) in Three or More Tumors.**
(DOCX)

Table S5 **Correlation of Contrast QC and QC Call Rate with Total CNA Count.**
(DOCX)
