# Supplementary material for: The Degree of Segmental Aneuploidy Measured by Total Copy Number Abnormalities Predicts Survival and Recurrence in Superficial Gastroesophageal Adenocarcinoma
Source: PLoS One. 2014 Jan 16;9(1):e79079. doi: 10.1371/journal.pone.0079079 (PMC3894223; doi:10.1371/journal.pone.0079079)
Supplement: Table S3 — Regions with Copy Number Gain (CN>3.025) in Three or More Tumors. (DOCX) [file pone.0079079.s003.docx]

**Supporting Table S3: Regions with Copy Number Gain (CN>3.025) in Three or More Tumors**

|  |  |  |  | **Previously Reported** | | |  |
| --- | --- | --- | --- | --- | --- | --- | --- |
| **Cytoband** | **Segment ID** | **Size (kbp)** | **Freq**  **N (%)** | **Deng, 2012** | **Gu, 2010** | **Dulak, 2012** | **Candidate Genes** |
| 1p31.1 | chr1: 72768926-72807263 | 38.3 | 4 (9.8) | Y* | N | N |  |
| 1p13.3 | chr1: 111377466-111379275 | 1.8 | 5 (12.2) | Y* | N | N |  |
| 1q21.3 | chr1: 152555351-152586177 | 30.8 | 3 (7.3) | Y* | N | Y* |  |
| 1q21.3 | chr1: 152762484-152768111 | 5.6 | 5 (12.2) | Y* | N | Y* |  |
| 1q23.1 | chr1: 158514204-158516040 | 1.8 | 6 (14.6) | Y* | N | N |  |
| 1q23.3 | chr1: 161616913-161617800 | 0.9 | 4 (9.8) | Y* | N | N |  |
| 2p22.3 | chr2: 34701648-34727699 | 26.1 | 4 (9.8) | N | N | N |  |
| 2q22.3 | chr2: 146864831-146866752 | 1.9 | 4 (9.8) | N | N | N |  |
| 2q24.3 | chr2: 164301733-164308144 | 6.4 | 3 (7.3) | N | N | N |  |
| 2q34 | chr2: 213190166-213191571 | 1.4 | 4 (9.8) | N | N | N | ERBB4 |
| 3p14.1 | chr3: 68728889-68747388 | 18.5 | 3 (7.3) | N | N | N |  |
| 3q22.3 | chr3: 137025641-137030836 | 5.2 | 3 (7.3) | Y* | N | N |  |
| 3q25.2 | chr3: 152344960-152351020 | 6.1 | 3 (7.3) | Y* | N | N |  |
| 3q26.2 | chr3: 168790740-169223435 | 432.7 | 5 (12.2) | Y* | Y† | Y† | MECOM |
| 4q12 | chr4: 55111660-55118158 | 6.5 | 3 (7.3) | N | N | N | PDGFRA |
| 4q13.1 | chr4: 64704724-64708168 | 3.4 | 3 (7.3) | N | N | N |  |
| 4q26 | chr4: 115174511-115181942 | 7.4 | 4 (9.8) | N | N | N |  |
| 4q32.2 | chr4: 161876877-161884592 | 7.7 | 4 (9.8) | N | N | N |  |
| 4q34.1 | chr4: 172372115-172379152 | 7.0 | 4 (9.8) | N | N | N |  |
| 5p15.2 | chr5: 13520784-13522465 | 1.7 | 3 (7.3) | Y* | N | N |  |
| 5p15.2–p14.3 | chr5: 14510271-20688655 | 6,178.4 | 6 (14.6) | Y* | N | N | Multiple (CDH18, MYO10, ANKH, FBXL7, …) |
| 5p13.3 | chr5: 31294338-31406493 | 112.2 | 4 (9.8) | Y* | Y† | N | CDH6 |
| 5q11.2 | chr5: 57327697-57335945 | 8.2 | 8 (19.5) | Y* | N | N |  |
| 6p22.1 | chr6: 27662601-27669093 | 6.5 | 4 (9.8) | Y* | N | N |  |
| 6p21.2–p21.1 | chr6: 38992638-41104919 | 2,112.3 | 7 (17.1) | Y* | N | N | Multiple (DNAH8, GLP1R, SAYSD1, …) |
| 6p21.1 | chr6: 41114945-44791135 | 3,676.2 | 6 (14.6) | Y* | Y* | Y | Multiple (VEGFA, …) |
| 6p12.3–6p12.2 | chr6: 46774413-52588342 | 5,813.9 | 4 (9.8) | Y* | N | N | Multiple |
| q14.1 | chr6: 78971746-79023585 | 51.8 | 4 (9.8) | N | N | N |  |
| 6q16.3 | chr6: 103736402-103760025 | 23.6 | 3 (7.3) | N | N | N |  |
| 7p14.1 | chr7: 38323495-38338275 | 14.8 | 13 (31.7) | Y* | N | N | TCRGC2, TARP |
| 7p11.2 | chr7: 54370032-55741552 | 1,371.5 | 8 (19.5) | Y | Y | Y | EGFR |
| 7q21.2–q21.3 | chr7: 91948478-96686530 | 4,738.1 | 5 (12.2) | Y | Y | Y | Multiple (CDK6, …) |
| 7q21.3 | chr7: 97123723-97593056 | 469.3 | 8 (19.5) | Y* | N | Y† | ASNS, TAC1 |
| 7q22.2 | chr7: 104467718-104482086 | 14.4 | 4 (9.8) | Y* | N | N | LHFPL3 |
| 7q31.1 | chr7: 109431898-109454105 | 22.2 | 7 (17.1) | Y* | N | N |  |
| 7q31.2 | chr7: 116402407-116421600 | 19.2 | 3 (7.3) | Y | N | Y | MET |
| 7q31.33 | chr7: 126045603-126046880 | 1.3 | 4 (9.8) | Y* | N | N |  |
| 8p23.3 | chr8: 31254-1235062 | 1,203.8 | 9 (22.0) | N | N | N | ERICH1, FBXO25 |
| 8p23.2 | chr8: 4136476-4140549 | 4.1 | 3 (7.3) | N | N | N | CSMD1 |
| 8p23.1 | chr8: 6465333-7798926 | 1,333.6 | 3 (7.3) | N | N | N | Multiple (defensins; SPAG11B) |
| 8q22.3 | chr8: 103055872-103060369 | 4.5 | 4 (9.8) | Y* | N | N | NCALD |
| 8p23.1 | chr8: 10497646-12242519 | 1,744.9 | 5 (12.2) | N | Y* | Y | Multiple (SOX7, BLK, GATA4, …) |
| 8q24.13 | chr8: 123061126-123073109 | 12.0 | 4 (9.8) | Y* | N | N |  |
| 8p23.1–p22 | chr8: 12420717-14295396 | 1,874.7 | 4 (9.8) | N | N | N | DLC1, LONRF1, MIR3926-1, -2, SGCZ |
| 8q24.13–q24.22 | chr8: 125856201-131786409 | 5,930.2 | 4 (9.8) | Y | Y | Y | Multiple (MYC, …) |
| 8q24.3 | chr8: 142145303-142894630 | 749.3 | 3 (7.3) | Y* | N | N | PTP4A3, GPR20 |
| 8p22 | chr8: 14425951-14727336 | 301.4 | 4 (9.8) | N | N | N | SGCZ, MIR383 |
| 9p24.1 | chr9: 5109992-5119782 | 9.8 | 3 (7.3) | N | N | N | JAK2 |
| 10p12.2 | chr10: 24374010-24377987 | 4.0 | 7 (17.1) | Y* | N | N | KIAA1217 |
| 10q11.22 | chr10: 47057251-47059396 | 2.1 | 6 (14.6) | N | N | N | ANXA8 |
| 11p15.4 | chr11: 4971186-4973664 | 2.5 | 5 (12.2) | N | N | N | None |
| 11q13.2–q13.4 | chr11: 68357918-71627544 | 3,269.6 | 8 (19.5) | Y | Y* | Y | Multiple (FGF3, FGF4, FGF19, CCND1, …) |
| 12p12.1–p11.22 | chr12: 24473641-28058626 | 3,585.0 | 6 (14.6) | Y | Y* | Y | Multiple ( KRAS, …) |
| 12q11 | chr12: 38053433-38134752 | 81.3 | 4 (9.8) | Y* | N | N |  |
| 12q24.13 | chr12: 112895543-113173458 | 277.9 | 7 (17.1) | N | N | N | RPH3A, PTPN11 |
| 13q13.3 | chr13: 35772328-36584783 | 812.5 | 3 (7.3) | Y* | N | N | DCLK1, NBEA |
| 13q14.2 | chr13: 48324142-48335395 | 11.3 | 5 (12.2) | Y* | N | N |  |
| 13q21.33–q31.1 | chr13: 72293307-83792511 | 11,499.2 | 6 (14.6) | Y | N | Y | Multiple: (KLF5, SPRY2, POU4F1, …) |
| 13q33.1 | chr13: 104276604-104279104 | 2.5 | 4 (9.8) | Y* | N | N |  |
| 14q32.31 | chr14: 101713411-101720665 | 7.3 | 5 (12.2) | N | N | N |  |
| 15q14 | chr15: 34176746-34177039 | 0.3 | 4 (9.8) | N | N | N | AVEN |
| 15q24.3 | chr15: 76884801-76898977 | 14.2 | 5 (12.2) | N | N | N | SCAPER |
| 16q23.1 | chr16: 76019857-76021326 | 1.5 | 3 (7.3) | N | N | N |  |
| 17q11.2 | chr17: 26078722-26674276 | 595.6 | 3 (7.3) | N | N | N | NLK |
| 17q12 | chr17: 37698485-38087513 | 389.0 | 8 (19.5) | Y | Y† | Y | HER2 |
| 17q21.2 | chr17: 38552785-39982446 | 1,429.7 | 11 (26.8) | N | N | N | Multiple (CDC6, TOP2A, RARA, …) |
| 18q11.2 | chr18: 19416638-23355313 | 3,938.7 | 4 (9.8) | Y | Y† | Y | Multiple (GATA6, LAMA3, …) |
| 20p13 | chr20: 1570953-1599031 | 28.1 | 6 (14.6) | Y* | N | N | SIRPB1 |
| 20q13.2 | chr20: 50359070-51999607 | 1,640.5 | 4 (9.8) | Y* | Y | N | ZPF64, TSHZ2 |
| 20q13.2 | chr20: 52675617-52767170 | 91.6 | 4 (9.8) | Y* | N | N | BCAS1 |
| 20q13.32–q13.33 | chr20: 57875482-58425657 | 550.2 | 3 (7.3) | Y* | N | N | PHACTR3, EDN3 |
| 20q13.33 | chr20: 59636681-62956154 | 3,319.5 | 3 (7.3) | Y* | N | N | Multiple ( PTK6, OGFR, …) |
| 22q13.1 | chr22: 39389666-39397409 | 7.7 | 3 (7.3) | N | N | N | APOBEC3B |

* There was a broad overlapping chromosomal region described or depicted in the cited publication.

† The cited publication described a nearby region (< 10 Mb).
