# Supplementary material for: The Degree of Segmental Aneuploidy Measured by Total Copy Number Abnormalities Predicts Survival and Recurrence in Superficial Gastroesophageal Adenocarcinoma
Source: PLoS One. 2014 Jan 16;9(1):e79079. doi: 10.1371/journal.pone.0079079 (PMC3894223; doi:10.1371/journal.pone.0079079)
Supplement: Table S4 — Regions with Copy Number Loss (CN<1.474) in Three or More Tumors. (DOCX) [file pone.0079079.s004.docx]

**Supporting Table S4: Regions with Copy Number Loss (CN<1.474) in Three or More Tumors**

|  |  |  |  | **Previously Reported** | | |  |
| --- | --- | --- | --- | --- | --- | --- | --- |
| **Cytoband** | **Location** | **Size (kbp)** | **Frequency**  **N (%)** | **Deng, 2012** | **Gu, 2010** | **Dulak, 2012** | **Candidate Gene(s)** |
| 1p31.1 | chr1: 72796039–72811794 | 15.8 | 3 (7.3) | N | N | N |  |
| 3p14.2 | chr3: 60282999–60665452 | 382.5 | 23 (56.1) | Y | Y | Y | FHIT |
| 4q26 | chr4: 115174511–115181942 | 7.4 | 4 (9.8) | Y* | N | N |  |
| 7p12.2 | chr7: 50418415–50430258 | 11.8 | 3 (7.3) | N | N | N |  |
| 7q36.3 | chr7: 157199488–158080383 | 880.9 | 6 (14.6) | N | N | Y | PTPRN2 |
| 7q36.3 | chr7: 157510150–157521798 | 11.6 | 4 (9.8) | N | N | Y | PTPRN2 |
| 9p24.3 - p24.1 | chr9: 46587–5107843 | 5,061.3 | 5 (12.2) | Y* | N | N |  |
| 9p24.3 - p24.2 | chr9: 1513213–3033537 | 1,520.3 | 4 (9.8) | Y* | N | N |  |
| 9p24.1 | chr9: 5030972–5093689 | 62.7 | 3 (7.3) | Y* | N | N | JAK2 |
| 9p24.1 - 9p21.3 | chr9: 6624512–22695986 | 16,071.5 | 11 (26.8) | Y | N | Y |  |
| 9p24.1 - p23 | chr9: 6631656–11692706 | 5,061.1 | 4 (9.8) | Y | N | Y | PTPRD |
| 9p21.3 | chr9: 21987305–22005076 | 17.8 | 6 (14.6) | Y | Y | Y | CDKN2A, 2B |
| 11q21 | chr11: 95075143–95861014 | 785.9 | 5 (12.2) | Y* | N | N | MAML2 |
| 11q21 | chr11: 95852605–95860330 | 7.7 | 4 (9.8) | Y* | N | N | MAML2 |
| 11q24.2–q25 | chr11: 124754991–131420805 | 6,665.8 | 7 (17.1) | Y* | N | Y | Multiple (CHEK1, ...) |
| 11q24.2 | chr11: 127200528–127203957 | 3.4 | 4 (9.8) | Y* | N | Y | BC030092 |
| 11q24.3 | chr11: 128349166–128354366 | 5.2 | 5 (12.2) | Y* | N | Y | ETS1 |
| 11q25 | chr11: 133079951–133088414 | 8.5 | 4 (9.8) | Y* | N | Y | OPCML |
| 12p13.31 | chr12: 9795227–9811725 | 16.5 | 3 (7.3) | Y* | N | N | LOC374443 |
| 12p11.1 | chr12: 34454852–34542819 | 88.0 | 3 (7.3) | Y* | N | N |  |
| 12q24.33 | chr12: 132126649–132133283 | 6.6 | 3 (7.3) | N | Y* | N | DB035218 |
| 15q13 | chr15: 26037137–26042230 | 5.1 | 3 (7.3) | N | N | N | ATP10A |
| 15q24.3 | chr15: 76884801–76902186 | 17.4 | 3 (7.3) | N | N | N | SCAPER |
| 16q23.1 | chr16: 78549222–79057257 | 508.0 | 9 (22.0) | Y | Y | Y | WWOX |
| 17p13.3 | chr17: 526–2316764 | 2,316.2 | 4 (9.8) | Y* | Y* | N | Multiple |
| 17p13.3 | chr17: 29513–133528 | 104.0 | 3 (7.3) | Y* | Y* | N | RPH3AL |
| 17p13.3 | chr17: 2118253–2152603 | 34.4 | 3 (7.3) | Y* | Y* | N | SMG6 |
| 18q11.2–q23 | chr18: 23585052–78015058 | 54,430.0 | 18 (43.9) | Y* | Y* | N | Multiple |
| 18q12.2 | chr18: 34874766–35138108 | 263.3 | 6 (14.6) | Y* | Y* | N | CELF4 |
| 18q12.3 | chr18: 38258716–38266706 | 8.0 | 7 (17.1) | Y* | Y* | N |  |
| 18q12.3–q21.1 | chr18: 42037737–43561082 | 1,523.3 | 8 (19.5) | Y* | Y* | N | Multiple |
| 18q21.2 | chr18: 48582103–51264612 | 2,682.5 | 7 (17.1) | Y* | Y* | Y | Multiple (SMAD4, DCC, …) |
| 18q21.33 | chr18: 60793956–60854769 | 60.8 | 8 (19.5) | Y* | Y* | N | BCL2 |
| 18q22.1–q22.2 | chr18: 63202918–67156570 | 3,953.7 | 10 (24.4) | Y* | Y* | N | Multiple |
| 20p12.1 | chr20: 15028375–15052480 | 24.1 | 3 (7.3) | N | Y† | Y | MACROD2 |
| 21q22.12 | chr21: 36255714–36375429 | 119.7 | 6 (14.6) | Y* | Y | Y | RUNX1 |
| 21q22.2 | chr21: 39947924–39953189 | 5.3 | 3 (7.3) | Y* | Y* | N | ERG |
| 21q22.3 | chr21: 45599677–48096958 | 2,497.3 | 3 (7.3) | Y* | Y* | N | Multiple (SUMO3, …) |
| 22q11.1–q11.21 | chr22: 17588702–20075772 | 2,487.1 | 4 (9.8) | Y* | Y* | N | Multiple (BID, …) |
| 22q11.22–q13.33 | chr22: 22486053–30510796 | 8,024.7 | 5 (12.2) | Y* | Y* | N | Multiple |
| 22q11.22–q13.33 | chr22: 30510796–51234456 | 20,723.7 | 9 (22.0) | Y* | Y* | N | Multiple |
| 22q13.2 | chr22: 41536901–41540855 | 4.0 | 5 (12.2) | Y* | Y* | N | EPS300 |
| 22q13.31 | chr22: 45550549–45569235 | 18.7 | 5 (12.2) | Y* | Y* | N | NUP50, LOC100506714 |
| 22q13.31 | chr22: 47953318–47964257 | 10.9 | 5 (12.2) | Y* | Y* | N |  |
| 22q13.32 | chr22: 48526817–48532341 | 5.5 | 5 (12.2) | Y* | Y* | N |  |
| 22q13.33 | chr22: 50963796–51160244 | 196.4 | 6 (14.6) | Y* | Y* | N | Multiple |
| Xp22.31 | chrX: 7407219–7449562 | 42.3 | 3 (7.3) | N | N | N |  |
| Xp21.1 | chrX: 31721525–31947413 | 225.9 | 10 (24.4) | N | Y | N | DMD |

* There was a broad overlapping chromosomal region described or depicted in the cited publication.

† The cited publication described a nearby region (< 10 Mb).
