# Supplementary material for: The Degree of Segmental Aneuploidy Measured by Total Copy Number Abnormalities Predicts Survival and Recurrence in Superficial Gastroesophageal Adenocarcinoma
Source: PLoS One. 2014 Jan 16;9(1):e79079. doi: 10.1371/journal.pone.0079079 (PMC3894223; doi:10.1371/journal.pone.0079079)
Supplement: Table S5 — Correlation of Contrast QC and QC Call Rate with Total CNA Count. (DOCX) [file pone.0079079.s005.docx]

## Supporting Table S5: Correlation of Contrast QC andQC Call Rate with Total CNA Count

|  | **Correlation with Total CNA Count** | | | |
| --- | --- | --- | --- | --- |
|  | **Spearman's rho** | **p-value** | **Pearson's r** | **p-value** |
| **Tumor contrast QC** | -.034 | .832 | .063 | .695 |
| **Tumor QC call rate** | .135 | .398 | .211 | .186 |
| **Normal contrast QC** | -.026 | .871 | .006 | .971 |
| **Normal QC call rate** | .204 | .201 | .169 | .290 |
